# A Five-Year Survey of Dematiaceous Fungi in a Tropical Hospital Reveals Potential Opportunistic Species

**DOI:** 10.1371/journal.pone.0104352

**Published:** 2014-08-06

**Authors:** Su Mei Yew, Chai Ling Chan, Kok Wei Lee, Shiang Ling Na, Ruixin Tan, Chee-Choong Hoh, Wai-Yan Yee, Yun Fong Ngeow, Kee Peng Ng

**Affiliations:** 1 Department of Medical Microbiology, Faculty of Medicine, University of Malaya, Kuala Lumpur, Malaysia; 2 Codon Genomics SB, Jalan Bandar Lapan Belas, Pusat Bandar Puchong, Selangor Darul Ehsan, Malaysia; Instituto de Higiene e Medicina Tropical, Portugal

## Abstract

Dematiaceous fungi (black fungi) are a heterogeneous group of fungi present in diverse environments worldwide. Many species in this group are known to cause allergic reactions and potentially fatal diseases in humans and animals, especially in tropical and subtropical climates. This study represents the first survey of dematiaceous fungi in Malaysia and provides observations on their diversity as well as *in vitro* response to antifungal drugs. Seventy-five strains isolated from various clinical specimens were identified by morphology as well as an internal transcribed spacer (ITS)-based phylogenetic analysis. The combined molecular and conventional approach enabled the identification of three classes of the Ascomycota phylum and 16 genera, the most common being *Cladosporium, Cochliobolus* and *Neoscytalidium*. Several of the species identified have not been associated before with human infections. Among 8 antifungal agents tested, the azoles posaconazole (96%), voriconazole (90.7%), ketoconazole (86.7%) and itraconazole (85.3%) showed *in vitro* activity (MIC ≤1 µg/mL) to the largest number of strains, followed by anidulafungin (89.3%), caspofungin (74.7%) and amphotericin B (70.7%). Fluconazole appeared to be the least effective with only 10.7% of isolates showing *in vitro* susceptibility. Overall, almost half (45.3%) of the isolates showed reduced susceptibility (MIC >1 µg/mL) to at least one antifungal agent, and three strains (one *Pyrenochaeta unguis-hominis* and two *Nigrospora oryzae*) showed potential multidrug resistance.

## Introduction

Dematiaceous fungi are a heterogeneous group of fungi with dark colonies and pigmented fungal elements. They are typically soil saprophytes, plant pathogens, and laboratory contaminants with a worldwide distribution in humid environments. Until 2008, more than 130 species from 70 genera have been recorded to be associated with infections in humans and animals [1], a vast increase from the 59 species belonging to 28 genera reported in 1996 by Rossmann *et al*. [2]. The genera most frequently involved in human infections include *Bipolaris*, *Curvularia*, *Exserohilum*, and *Alternaria* [3]. Many of the fungi are common allergens growing indoors. Besides causing hypersensitivity reactions in susceptible individuals that sometimes lead to acute exacerbation of asthma, they are also important opportunistic pathogens in immunocompromised patients [4], [5]. Although many of the cutaneous, subcutaneous, and corneal infections associated with dematiaceous fungi have been reported to be common in tropical and subtropical countries [6], to our knowledge, there has not been any extensive report on the diversity and *in vitro* antifungal susceptibility patterns of dematiaceous fungi isolated from clinical samples in a tropical country like Malaysia.

The spectrum of diseases associated with dematiaceous fungi ranges from superficial skin and soft tissue infections to disseminated sepsis with high mortality. The most common infections are phaeohyphomycosis [7], chromoblastomycosis [8], and eumycetoma [9], [10]. In a retrospective analysis of 101 cases of central nervous system (CNS) phaeohyphomycosis, over half occurred in immunocompetent patients [11]. Chromoblastomycosis is mainly associated with *Fonsecaea*, *Phialophora*, *Cladosporium*, *Exophiala* and *Rhinocladiella* species [8]. Eumycetoma is caused primarily by *Madurella mycetomatis*, but the aetiological agents of this disease vary with geographical regions [9]. In the tropics, *Curvularia* species are significant causes of fungal keratitis associated with trauma from fungus-contaminated plant materials [12], [13] while the *Bipolaris*, *Curvularia*, *Exserohilum*, *Alternaria* and *Drechslera* are frequently reported to be involved in invasive sinusitis [14]–[17]. Systemic dematiaceous fungal infections are rare compared to systemic candidiasis and aspergillosis. However, dematiaceous fungi are being increasingly recognized as invasive human pathogens [18] especially in organ transplant recipients [7].

The identification of dematiaceous fungi is traditionally based on the observation of differentiating morphological structures such as annellides or phialides, the presence or absence of collarettes on adelophialides, the differentiation of conidiophores, and septation of macroconidia. Molecular tests that are available today offer an alternative approach to the identification of dematiaceous fungi [19], [20]. The molecular test strategy most often used is DNA amplification, followed by sequence analysis of variable regions within pan-fungal conserved genes (18S rRNA, 5.8S rRNA and the 5′ end of the 28S rRNA) or the internal transcribed spacers (ITS1 and ITS2) [21]–[23]. In the past decade, the ITS1-5.8S-ITS2 region has become a useful alternative diagnostic tool for the identification of fungi of agricultural and clinical importance [24], [25].

The aim of this study was to appraise the diversity of dematiaceous fungi isolated from patients with signs and symptoms of fungal infection and the antifungal drug susceptibility profiles of these isolates. The information from this survey could be useful for the formulation of appropriate drug therapy for patients with suspected fungal infections in a tropical setting.

## Materials and Methods

### Ethics Statement

This study involved only the phenotypic and phylogenetic analysis of dematiaceous fungi isolated from routine cultures in the mycology laboratory. As no information was used that could lead to patient identification, it was considered unnecessary to apply for approval from the university's Medical Ethics Committee (http://www.ummc.edu.my/index.php/2011-09-28-08-46-26/2011-10-03-03-14-40/158-ummc-medical-ethics).

### Sample Collection and Processing

The fungal isolates examined in this study were obtained from clinical specimens collected from patients attending the University Malaya Medical Centre (UMMC), Malaysia. Skin scrapings and nail clippings were collected from patients with suspected dermatomycosis. Respiratory specimens were routinely screened for fungal pathogens in patients presenting with respiratory tract infection. Other tissue fluids and tissues were processed for fungal isolation only on request by physicians when patients had clinical manifestations of fungal infection. All specimens were processed according to the laboratory's standard operating procedures (SOP). Direct microscopic examinations were performed on skin scrapings, hair and nail clippings treated with 40% potassium hydroxide (KOH), and on tissue smears after staining with Gram and Gomori's methenamine-silver nitrate stains. Cultures were put up on Sabouraud Dextrose Agar (SDA) with chloramphenicol (0.25 g/mL) and sheep blood agar. Blood specimens were placed into BD BACTEC Myco/F Lytic Medium for incubation in the BD BACTEC 9240 Blood Culture System (Becton Dickinson, USA). Positive blood samples were sub-cultured onto SDA with chloramphenicol and sheep blood agar. Swabs and nasopharyngeal secretions were inoculated directly onto SDA with chloramphenicol and sheep blood agar.

### Fungal Isolates

Fungal isolates were grown on SDA incubated at 30°C for up to 4 weeks with alternate day examination for growth. When mixed colonies were observed, each colony type was sub-cultured for purity. Each fungal culture was observed macroscopically for colonial characteristics such as colour, texture, and topology. Tease mounts and slide cultures were carried out to study the arrangement of conidia under the light microscope. Slide cultures were prepared by growing the fungi on Potato Dextrose Agar (PDA) to encourage mould sporulation for 7 to 14 days at 30°C. The slides were examined periodically, with lactophenol cotton blue staining carried out when sufficient growth was attained.

### DNA Extraction

Pure cultures on SDA plates were harvested by flooding each plate with 3 mL of phosphate buffered saline (PBS, pH 7.4) followed by gentle scraping of the agar surface with an L-shaped glass Pasteur pipette. The suspension was then collected into a 15 mL centrifuge tube with PBS washed glass beads and vortex-mixed for 5 min. For DNA extraction, 200 µL of suspension was processed with the ZR Fungal/Bacterial DNA MiniPrep™ (Zymo Research, USA) according to the protocol provided by the manufacturer.

### PCR and Sequencing

For the amplification of the ITS1-5.8S-ITS2 region, the ITS1 (5'- TCC GTA GGT GAA CCT GCG G -3') and ITS4 (5'- TCC TCC GCT TAT TGA TAT GC -3') primer sets were used as forward and reverse primers respectively [26]. The 20 µL reaction volume contained 0.2 µM of each primer, 0.2 mM of deoxynucleotides, 1.5 mM of MgCl_2_, 1X PCR buffer, and 1 unit of DyNAzyme™ EXT DNA polymerase (Finnzymes, Finland) together with 5 µL extracted DNA. The PCR parameters consisted of an initial denaturation at 95°C for 5 min, followed by 30 cycles of denaturation at 94°C for 30 seconds, annealing at 58°C for 30 seconds, extension at 72°C for 1 min, and a final extension at 72°C for 10 min. The PCR product was then electrophoresed in 1% (w/v) agarose gel at 120 V for 30 min, purified and sent for Sanger sequencing (1st Base Laboratories Kuala Lumpur, Malaysia). Base and quality calling of sequenced ITS was performed using TraceTuner v3.0.6 [27]. Lucy version 1.20 [28] and the included zapping.awk script was then run to trim low quality bases (Phred value <20) called by TraceTuner from both ends of the sequences. Cleaned sequences with complete ITS1-5.8S-ITS2 regions and consensus sequences of duplicated ITS copies were subjected to further analysis. The sequences obtained in this study were deposited in GenBank under accession numbers shown in Table S1.

### Data Mining

Unique ITS nucleotide sequences with complete ITS1-5.8S-ITS2 regions were searched against National Centre for Biotechnology Information (NCBI, US) non-redundant database using local Nucleotide-BLAST program to identify the molecularly-related isolates. To avoid false identification due to the errors deposited in NCBI GenBank database, we collected the top five hits from the blast results. ITS sequences of all species collected were then randomly mined from the NCBI GenBank for their complete ITS sequences with at least two sequences verified for each species, except when there was only one record in the database.

### Phylogenetic Analysis

All ITS sequences from clinical isolates, together with those retrieved from the NCBI database and two out-group strains of *Saccharomyces boulardii*, were subjected to phylogenetic analysis. Multiple sequence alignment of all data mined ITS sequences was generated using M-Coffee [29] which adopted other packages to compute the alignments and used T-Coffee to combine all these alignments into one unique final alignment. Phylogenetic analysis was then performed using MrBayes [30]. Bayesian MCMC analysis was started by sampling across the entire general time reversible (GTR) model space. A total of 600,000 generations were run with a sampling frequency of 100, and diagnostics were calculated every 1,000 generations. A burn-in setting of 25% was used to discard the first 1,500 trees.

### Antifungal Susceptibility Testing

The Epsilometer Test (Etest, Biomerieux, France) for antifungal susceptibility was carried out to determine the minimum inhibitory concentration (MIC) of antifungal drugs according to the manufacturer's protocol. The antifungal drugs tested in this study were amphotericin B (AMB), five azoles, *viz*. itraconazole (ITC), voriconazole (VRC), ketoconazole (KTC), fluconazole (FLC), and posaconazole (PSC), and two echinocandins, *viz*. anidulafungin (ANID) and caspofungin (CAS).

## Results

### Fungal Isolates

A total of 75 (6%) black fungi were identified from a collection of 1,250 molds isolated in the Mycology Unit of UMMC in a 5-year period, from 2006 to 2011. These isolates were mostly obtained from superficial skin, nail, subcutaneous and nasopharyngeal specimens (62), blood (10), and tissue biopsies (3) (Table S1). All isolates formed typical dark brown, olivaceous or black colonies, appearing dark on the reverse side of the agar plate and displaying septate fungal elements under the microscope.

### Morphological Identification

**Figure 1:**
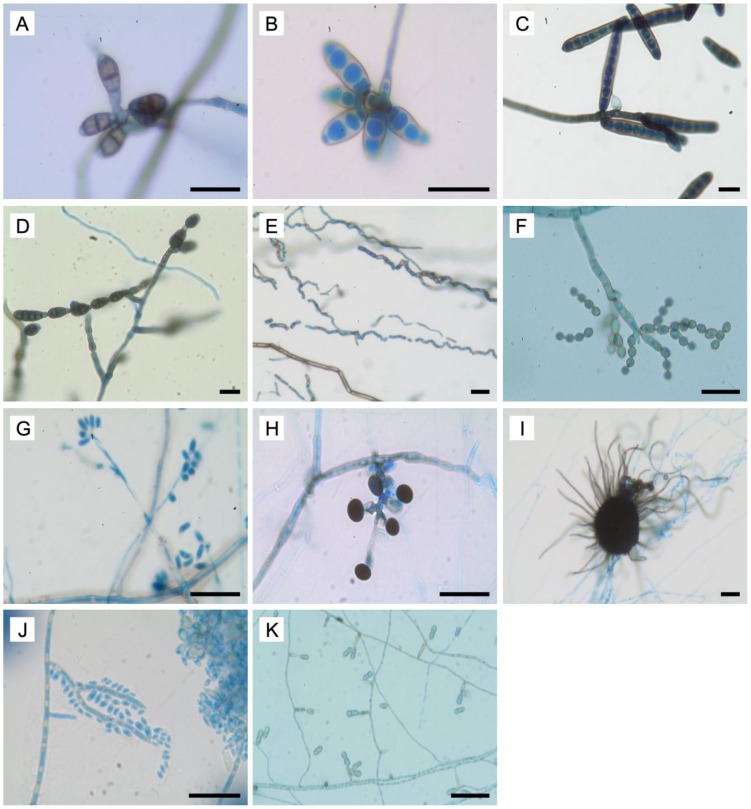
Microscopic features of dematiaceous fungi. (A-D) Macroconidia of *Curvularia*, *Bipolaris*, *Exserohilum* and *Alternaria* (E) Arthroconidia of *Neosyctalidium* (F) Globose chain conidia and ramoconidia of *Cladosporium* (G) conidia of *Daldinia* (H) Dark conidia of *Nigrospora* (I) *Chaetomium* perithecium covered with long setae and dark ascospores (J) Spine-like conidiophore and hyaline conidia of *Exophiala* (K) *Ochroconis* two-celled clavate conidia with cylindrical conidiophore. *Bars* 20 µm.

Identification based on morphological characteristics enabled the classification of most (89.3%) of the dematiaceous fungi at the genus level, using the criteria previously described [19], [20], [31], [32] (Figure 1). Generally, *Bipolaris*, *Curvularia* and *Exserohilum* were characterized by floccose and brown to black colonies. The macroconidia of *Curvularia* had thin cell walls and transverse septa. They often appeared as curved structures due to the swelling of the central cell, which was darker compared to the end cells. The macroconidia were thick-walled and fusiform, with three to four septations in *Bipolaris*, and cylindrical in shape with seven to eleven septa in *Exserohilum*. *Alternaria* colonies were greenish black with short, woolly hyphae. The conidia of this genus were longitudinally and transversely septate, brown and ovate arranged singly or in chains. Members of *Neoscytalidium* produced colonies that were densely fluffy with gray to dark gray aerial mycelia. These fungi were typified by arthroconidia arranged in chains in a zig-zag appearance. *Cladosporium* isolates were velvety with olivaceous green to black green colonies. Their characteristic features included conidiophores that were straight and branched at the apical region, conidia that were ovoid to globose, with dark scarring and arranged in chains, and ramoconidia.

*Daldinia* colonies appeared felty and whitish. Microscopically, the conidiophores were irregularly branched with conidiogeneous cells arising from the terminus, and conidia were ellipsoid. *Nigrospora* colonies were wooly and white, becoming black on aging. A single dark conidium which was spherical or oblate with a smooth wall was found at the apex of conidiogeneous cells. *Chaetomium* was characterized by dark colored perithecium with ostiolate and covered with long setae. The ascospores observed were mostly ovoidal, dark- colored and single-celled. The colonies were white with aerial mycelium, becoming grayish when mature. The *Exophiala* colonies were olivaceous-black, mucoid at the center and greenish aerial mycelium were observed at the periphery of aged colonies. Conidiophores were simple or branched, erect and spine-like with one-celled, hyaline, subglobose to ellipsoidal conidia. Lastly, the colonies of *Ochroconis* were dry, red-brown with red-brown pigment diffused into the growth medium. The conidiophores were cylindrical, bearing two-celled, light brown and clavate conidia.

Morphological identification was, however, not possible when there were overlapping characteristics between genera, and for strains that did not sporulate. Five mycelia sterilia and three other isolates (UM 221, UM 238 and UM 1110) could not be identified by their morphological features alone.

### Phylogenetic Analysis and Taxonomic Classification

**Figure 2:**
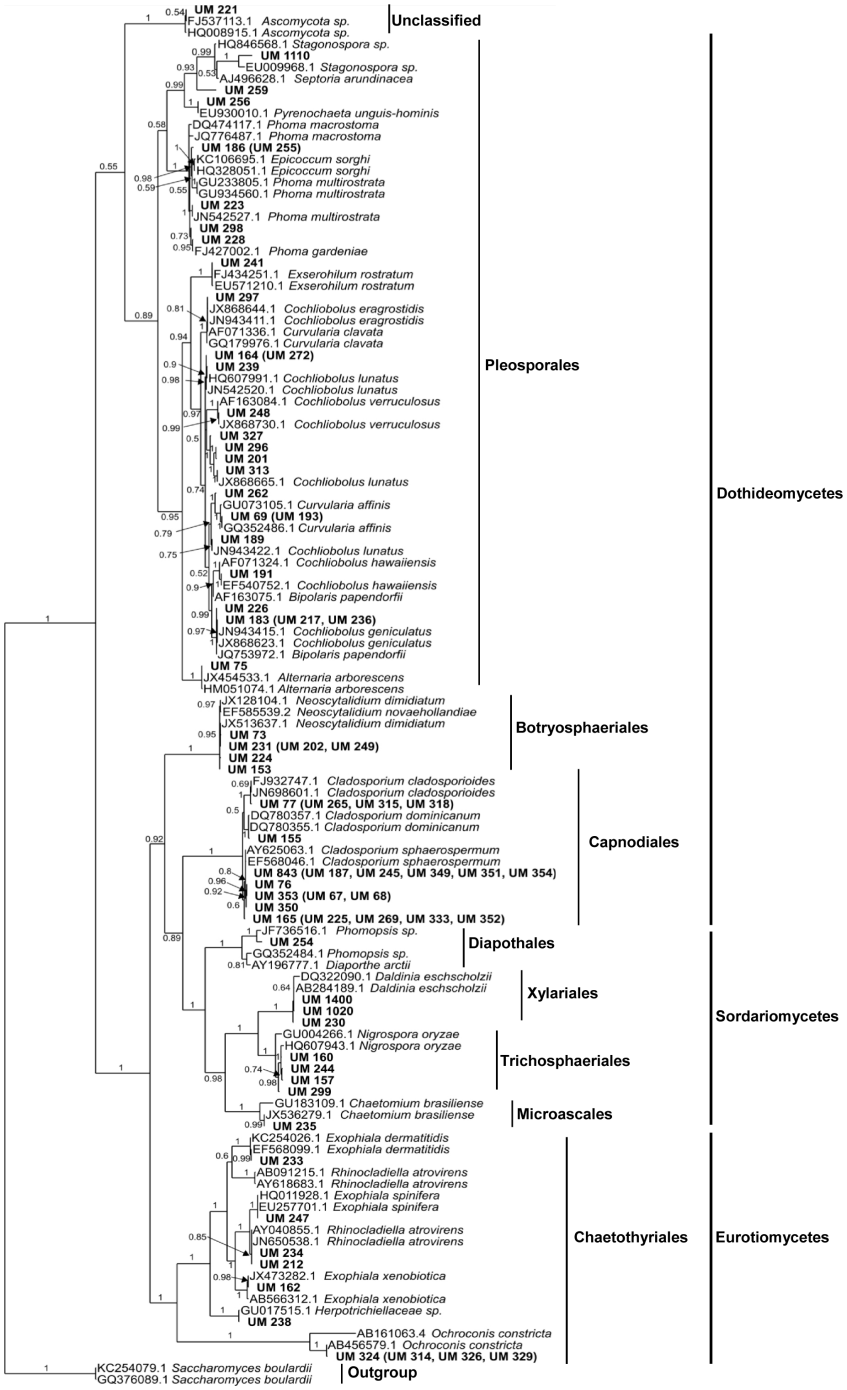
Classifications of fungal isolates. Bayesian tree generated with general time reversible (GTR) model space based on unique ITS1-5.8S-ITS2 gene sequences with two strains of *Saccharomyces boulardii* as out-group. Isolate sequence duplicates are listed in parentheses next to their representative. Clinical isolates from UMMC used in this study are printed in bold. Bayesian posterior probability values for every clustering are printed on each node.

A total of 120 ITS sequences were used for the Bayesian tree construction (Figure 2). These comprised 41 unique sequences from UM isolates, 10 representative sequences from 34 UM isolates with shared sequences, 67 from reference dematiaceous fungi and two out-group strains of *Saccharomyces boulardii*. The phylogenetic tree resolved the 75 UM isolates into four distinct classes of Dothideomycetes (74.7%), Sordariomycetes (12.0%), Eurotiomycetes (13.3%), and one unclassified cluster made up of UM 221 and two reference Ascomycota species. Each class encompassed one to four members of orders comprising one to 28 UM isolates. Most of the isolates (65.3%) were in the two largest orders Pleosporales (28) and Capnodiales (21). From their clustering with reference strains, the majority of the isolates (86.7%) were resolved to the species level.

Congruence between morphological and molecular identification was observed for all *Cladosporium*, *Neoscytalidium*, *Ochroconis*, *Daldinia* and *Nigrospora* isolates (Table S1). Among the incongruent classifications were six of seven *Bipolaris* spp. and seven of 11 *Curvularia* spp., all resolved in the phylogenetic analysis as *Cochliobolus* spp.. UM 256 was identified as *Phoma* by morphology but *Pyrenochaeta* by molecular analysis, while UM 212 and UM 234 were two isolates with morphological features of *Exophiala* species clustered with *Rhinocladiella atrovirens* in the phylogenetic tree. Of the five mycelia sterilia, only one was identified as *Nigrospora oryzae*; the other 4 remained unclassified even after molecular analysis. Similarly, of the three isolates with no identifiable morphological features, UM 238 was resolved as a *Herpotrichiellaceae* sp., UM 221 showed 100% ITS sequence similarity with *Ascomycota* sp. FJ537113, while UM 1110 was found to be closely related to *Stagonospora* sp. or *Septoria arundinacea*.

### Antifungal Susceptibility Testing

The results from the *in vitro* antifungal susceptibility tests varied among the various genera and species of dematiaceous fungi in this study (Table S1). As currently, there are no established guidelines on MIC breakpoints for dematiaceous fungi, interpretive comments in this study are based upon an MIC of ≤1 µg/mL being considered an indicator of potential susceptibility for most of the drugs used to treat infections by dematiaceous fungi [3]. Going by this guideline, PSC showed the highest *in vitro* activity (96% with MIC ≤1 µg/mL), followed by VRC (90.7%), ANID (89.3%), KTC (86.7%), ITC (85.3%), CAS (74.7%) and AMB (70.7%). FLC appeared to be the least active with 10.7% of isolates showing potential susceptibility and 34.7% having MIC>256 µg/mL. Among the isolates, *Pyrenochaeta unguis-hominis* and *N. oryzae* showed reduced susceptibility (MIC >1 µg/mL) to the largest number of antifungals with the former showing potential susceptibility to only ANID (Table 1). Overall, 45.3% of isolates showed reduced susceptibility to at least one antifungal.

**Table 1:** *In vitro* susceptibility of dematiaceous fungal isolates to antifungal agents, grouped according to MIC^a^ categories.

Fungal Identity, (n^b^)	Antifungal Drugs and MIC^a^ Categories																							
	AMB^c^			KTC^d^			FLC^e^			ITC^f^			VRC^g^			PSC^h^			ANID^i^			CAS^j^		
	A	B	C	A	B	C	A	B	C	A	B	C	A	B	C	A	B	C	A	B	C	A	B	C
*Alternaria arborescens* (1)	1			1					1	1			1			1			1			1		
*Ascomycota* sp. (1)	1				1				1		1		1			1			1					1
*Chaetomium brasiliense* (1)		1		1				1		1			1			1			1			1		
*Cladosporium cladosporioides* (4)	3	1		4			1		3	4			4			4			4					4
*Cladosporium dominicanum* (1)		1		1					1		1			1		1			1			1		
*Cladosporium sphaerospermum* (16)	6	9	1	14	2		1	7	8	15	1		15	1		16			15	1		14	2	
*Bipolaris papendorfii/Cochliobolus geniculatus* (1)	1			1				1		1			1			1			1			1		
*Cochliobolus geniculatus (3)*	3			3				3		3			3			3			3			3		
*Cochliobolus hawaiiensis* (1)	1			1				1		1			1			1			1			1		
*Cochliobolus lunatus* (8)	8			8			2	5	1	8			7	1		8			8			8		
*Cochliobolus verruculosus* (1)	1			1				1		1			1			1			1			1		
*Curvularia affinis* (3)	3			3				3		3			3			3			3			3		
*Curvularia eragrostidis* (1)	1				1				1		1		1			1			1			1		
*Daldinia eschscholzii* (3)	3			3			1	2		3			3			3			3			1	2	
*Exophiala dermatitidis* (1)	1			1				1		1			1			1					1			1
*Exophiala spinifera* (1)	1			1				1		1			1			1					1			1
*Exophiala xenobiotica* (1)	1			1				1		1			1			1			1					1
*Exserohilum rostratum* (1)	1			1				1		1			1			1			1			1		
*Herpotrichiellaceae* sp. (1)	1			1				1		1			1			1			1			1		
*Neoscytalidium dimidiatum* (4)	4			4			1	3		3	1		4			4			4			4		
*Neoscytalidium dimidiatum/Neoscytalidium novaehollandiae* (2)	2			2			1	1		1	1		2			2			2			2		
*Nigrospora oryzae* (4)	4			2	1	1		2	2	1	1	2	1	3		2		2		3	1		1	3
*Ochroconis constricta* (4)			4	3		1			4	4			4			4			4			4		
*Phoma gardeniae* (2)		2		2				2		2			2			2			2			2		
*Phoma multirostrata* (1)	1			1				1		1			1			1			1			1		
*Phoma multirostrata*/*Epicoccum sorghi* (2)	2			2			1	1		2			2			2			2			2		
*Phomopsis sp./Diaporthe arctii* (1)	1			1					1			1	1			1			1			1		
*Pyrenochaeta unguis-hominis* (1)		1				1			1			1			1			1	1					1
*Rhinocladiella atrovirens* (2)		2			2				2	2			2			2			1		1	1		1
*Stagonospora* sp.*/Septoria arundinacea* (2)	2			2				2		2			2			2			2			1		1
**Total**	**53**	**17**	**5**	**65**	**7**	**3**	**8**	**41**	**26**	**64**	**7**	**4**	**68**	**6**	**1**	**72**	**0**	**3**	**67**	**4**	**4**	**56**	**5**	**14**

a minimum inhibitory concentration.

b Number of fungal isolates.

c Amphotericin B.

d Ketoconazole.

e Fluconazole.

f Itraconazole.

g Voriconazole.

h Posaconazole.

i Anidulafungin.

j Caspofungin.

MIC categories:

Category A: ≤1 µg/mL (FLC: ≤1 µg/mL).

Category B: >1–32 µg/mL (FLC: >1–256 µg/mL).

Category C: >32 µg/mL (FLC: >256 µg/mL).

### Clinical Significance of Isolates

The ubiquitous presence of dematiaceous fungi in the environment makes it very difficult to gauge their clinical significance when they are isolated from patient samples. In this study, we believe the majority of our isolates are not contaminants or colonizers as all isolates were from symptomatic patients, most of whom had a physician's diagnosis of fungal infection or were started on antifungal therapy. Moreover, for superficial skin and nail samples, positive cultures correlated well with signs of fungal proliferation or tissue invasion under direct KOH microscopic examination.

## Discussion

With increasing recognition of the important role of fungi in human infections, diagnostic laboratories are now expected to be able to rapidly detect and accurately identify fungal pathogens to ensure early and appropriate therapy for infected patients. We have utilized the ITS sequence-based phylogenetic analysis in the classification of most of the dematiaceous fungi in this study, with good congruence attained with morphological identification. The molecular species differentiation was particularly useful for the fungi with ambiguous microscopic features, such as *Phoma* and *Pyrenochaeta*, two coelomycetes with overlapping pycnidial, conidial and cultural characteristics that were difficult to distinguish [3], [33], [34]. In the Chaetothyriales order, the synanamorphs of *Exophiala* and *Rhinocladiella* make the molecular approach a better option for their identification [35], [36]. Most of the *Bipolaris* and *Curvularia* species were resolved as *Cochliobolus*, the teleomorph of *Bipolaris* and *Curvularia* [37], [38]. Nevertheless, there were five isolates not resolved to the genus or species level by the ITS sequence analysis due to insufficient information in the current GenBank database [39]. More extensive sampling and further studies using multi-locus phylogeny would improve the identification of these dematiaceous fungi in the future.

Some of the dematiaceous fungi isolated in this study have been previously reported to cause human infections [3], [40]–[42]. Ten of the 16 genera we identified are listed as potential aetiological agents of phaeohyphomycosis in the guidelines compiled by an expert panel of the European Society of Clinical Microbiology and Infectious Diseases (ESCMID) and European Confederation of Medical Mycology (ECMM) [43] (Table 2). Interestingly, we encountered seven species that, to our knowledge, have not been reported to be associated with human infections. These are *Cladosporium dominicanum*, *Curvularia affinis* and *Ochroconis constricta* (from skin scrapings), *Curvularia eragrostidis* (from nails), *Daldinia eschscholzii* (from skin, nails and blood samples), *Phoma gardeniae* (from skin lesion swab and nails), and *Stagonospora* sp./*S. arundinacea* (from nasopharyngeal secretion and skin scrapings). The possibility that these clinical isolates might be contaminants or had been due to the patients' incidental exposure to fungal spores cannot be totally discounted. However, in all these cases, no other pathogens that could account for the patients' signs and symptoms were identified at the infected sites.

**Table 2:** Genera and MIC^a^ profiles of potential phaeohyphomycosis aetiological agents compiled by Chowdhary *et al*. [43] and isolates identified in this study.

Genus	Fungal species	Source	Case reported	MIC^a^ (µg/mL)				
				AMB^b^	FLC^c^	ITC^d^	VRC^e^	PSC^f^
*Chaetomium*	*C. brasiliense*	this study	[44]	12	4.0	0.5	0.012	0.75
	*C. globosum*	[43]	-	0.5–8.0	32->64	0.03–0.5	0.5	-
	*C. perlucidum*	[43]	-	0.25	-	0.01–0.06	0.5	0.06–0.12
*Bipolaris*	*Cochliobolus hawaiiensis*	this study	[19], [45]	0.016	1.5	0.012	0.023	0.008
	*B. hawaiiensis*	[43]	-	0.12–0.25	>64	0.03–0.5	0.25–2.0	0.03–0.5
	*B. australiensis*	[43]	-	0.06–0.12	8.0–16.0	0.25–0.5	0.05–1.0	0.06
	*B. spicifera*	[43]	-	0.03–4.0	4.0->64.0	0.03–8.0	0.25–4.0	0.03–2.0
	*B. papendorfii/Cochliobolus geniculatus*	this study	[19], [46]	0.012	1.0	0.012	0.023	0.006
*Curvularia*	*Cochliobolus geniculatus (C. senegalensis)*	this study	[19], [47]	<0.002–0.094	1.5–8	0.004–0.125	0.016–0.064	0.012–0.032
	*Cochliobolus lunatus*	this study	[19], [48]	0.003–0.064	0.38->256	<0.002–0.5	0.008–1.0	0.003–0.19
	*Cochliobolus verruculosus*	this study	[19]	0.047	1.5	0.004	0.016	0.012
	*C. senegalensis*	[43]	-	0.06–0.50	2.0–16.0	0.06–1.0	0.12–4.0	0.03–0.50
	*C. lunata*	[43]	-	0.12->16.0	>64	0.12->16	0.25–1.0	0.03–0.5
	*Curvularia spp.*	[43]	-	0.06->16.0	64->64	0.03->16	0.15->16	0.03–4.0
*Exophiala*	*E. dermatitidis*	this study	[19], [49]	0.5	24.0	0.38	0.032	0.094
	*E. spinifera*	this study	[19], [50], [51]	0.047	2.0	0.064	0.032	0.018
	*E. xenobiotica*	this study	[52], [53]	0.064	6.0	<0.002	0.016	0.016
	*E. dermatitidis*	[43]	-	0.01–0.5	-	0.03–0.5	0.06–1.0	0.03–0.25
	*E. spinifera*	[43]	-	0.25–4.0	0.12->64	0.01–0.12	0.06–1.0	0.01–0.06
	*E. jeanselmei*	[43]	-	0.25–2.0	8.0–32.0	0.01–0.25	0.06–2.0	0.01–0.06
*Exserohilum*	*E. rostratum*	this study	[19], [54]–[57]	0.047	32.0	0.125	0.25	0.012
	*E. rostratum*	[43]	-	0.03–0.12	-	0.03–0.12	0.03–1.0	0.03–0.12
Neoscytalidium	*N. dimidiatum*	this study	[31], [58]	0.008–0.064	0.125–16	0.003–12	0.016–0.064	0.003–0.125
	*N. dimidiatum/N. novaehollandiae*	this study	-	0.032	0.19–3	0.032; 1	<0.002–0.003	0.008–0.047
	*N. dimidiatum*	[43]	-	0.06–1.0	-	0.03->16	0.03–4	0.06–32
*Ochroconis*	*O. constricta*	this study	[59]	>32	0.38->256	0.024–0.25	0.19–0.75	0.047–0.25
	*O. gallopava*	[43]	-	0.12–1.0	16.0->64.0	0.01–0.50	0.12–2.0	0.01–0.12
	*O. tshawytschae*	[43]	-	4	>64.0	0.5	0.12	-
Phoma	*Phoma multirostrata*	this study	[60]	0.75	8.0	0.094	0.064	0.19
	*Phoma multirostrata*/*Epicoccum sorghi*	this study	[19]	0.016–0.023	0.19->256	0.19–0.25	0.064–0.125	0.125–0.19
	*Phoma spp.*	[43]	-	0.5-1.0	-	0.25–8.0	0.25–8.0	-
Pyrenochaeta	*P. unguis-hominis*	this study	[19], [40]	1.5	>256	>32	>32	>32
	*P. romeroi*	[43]	-	4	>64	0.5	4	0.5
Rhinocladiella	R. atrovirens	this study	[20], [61], [62]	8	>256	0.19–0.38	0.5–0.75	0.008–0.047
	*R. aquaspersa*	[43]	-	1.0–2.0	32–64	0.06–0.12	2	0.06–0.12
	*R. mackenziei*	[43]	-	1.0->16.0	16.0->64.0	0.01–0.25	0.01–2.0	0.01–0.25

The cases reported are from de Hoog *et al*. 2000 [19], Ellis *et al*. 2007 [20], Khan *et al*. 2009 [31], Hubka *et al*. 2011 [44], Mikosz *et al*. 2014 [45], Da Cunha *et al*. 2012 [46], Guarro *et al*. 1999 [47], Carter and Boudreaux 2004 [48], Oztas *et al*. 2009 [49], Rajendran *et al*. 2003 [50], Wang *et al*. 2013 [51], Aoyama *et al*. 2009 [52], Morio *et al*. 2012 [53], Hsu and Lee 1993 [54], Andes and Casadevall 2013 [55], Pappas *et al*. 2013 [56], Saint-jean *et al*. 2007 [57], Mani *et al*. 2008 [58], Malani *et al*. 2001 [59], Singh and Barde 1990 [60], English 1980 [40], Del Palacio-Hernanz *et al*. 1989 [61], and Rajput *et al*. 2011 [62].

a minimum inhibitory concentration.

b Amphotericin B.

c Fluconazole.

d Itraconazole.

e Voriconazole.

f Posaconazole.

The development of antifungal susceptibility testing is relatively recent. Various methods are now available to assess antifungal properties [63] but these have been used mostly on common fungal pathogens such as *Candida* and *Aspergillus* spp. [3]. The Clinical and Laboratory Standards Institute (CLSI) has guidelines for both broth microdilution [64] and disk diffusion [65] testing of filamentous fungi that cause cutaneous and invasive fungal infections. However, the interpretation of results is still problematic as no reliable breakpoints have been published for mold MICs. In the general guidelines recently proposed for a limited number of antifungals [66], molds are considered susceptible to AMB, ITC, PSC, VRC, and CAS when the MIC is ≤1 µg/mL, intermediate when the MIC is 2 µg/mL, and resistant when the MIC is ≥4 µg/mL. It is not known whether these guidelines can be applied to dematiaceous fungi. In this study, we used the Etest which is a commercial gradient diffusion test that has been introduced to facilitate antifungal testing in diagnostic laboratories. This test has been shown to correlate well with the CLSI methods in the testing of yeasts [67] and *Aspergillus* spp. [68], [69] against azoles and echinocandins. In the absence of established MIC breakpoints for dematiaceous fungi, we categorized the MICs obtained for our isolates arbitrarily, to indicate potential susceptibility (MIC ≤1 µg/mL) and two levels of potential resistance (>1–32 µg/mL or >1–256 µg/mL, and >32 or >256 µg/mL). The results showed ITC, PSC and VRC to have the best *in vitro* activity against all the dematiaceous fungi tested. In contrast, more than a third of the isolates had high MICs (>256 µg/mL) with FLC. These observations are consistent with previous reports on the enhanced antifungal activity of the later generation triazoles [70], [71] and the limited activity of FLC on molds [72]. Similarly, CAS which had been previously reported to be less active against filamentous fungi other than *Aspergillus* spp. [73] was shown in our study to be less active on dematiaceous fungi than ANID and the azoles other than FLC. Our results support the recommendation by the ESCMID and ECMM to use ITC, VRC, and PSC as the treatment of choice for phaeohyphomycosis [43]. However, it is also evident that there is considerable variation in the pattern of potential resistance among the diverse species of dematiaceous fungi; hence, the choice of antifungal agents for therapy should, as far as possible, be based on the results of antifungal susceptibility testing in individual cases.

## Conclusions

Many dematiaceous fungi are associated with invasive human infections. In this study, we have successfully used ITS-based phylogenetic analysis in conjunction with morphological characteristics to resolve and classify the dematiaceous fungi isolates from a Malaysian hospital. We have also demonstrated a congruence of results from the ITS-based technique with those by morphological traits. A combination of both approaches, together with antifungal drug susceptibility testing, would greatly aid in the rapid identification of these fungal species as well as in determining the appropriate therapeutic options for human infections caused by dematiaceous fungi.

## Supporting Information

Table S1 **Isolation strain, clinical source, morphological identity, molecular identity based on phylogenetic analysis, accession number in GenBank, and minimum inhibitory concentration (MIC) data of 75 UM isolates of dematiaceous fungi.**
(DOCX)
