# Supplementary material for: A Five-Year Survey of Dematiaceous Fungi in a Tropical Hospital Reveals Potential Opportunistic Species
Source: PLoS One. 2014 Aug 6;9(8):e104352. doi: 10.1371/journal.pone.0104352 (PMC4123927; doi:10.1371/journal.pone.0104352)
Supplement: Table S1 — Isolation strain, clinical source, morphological identity, molecular identity based on phylogenetic analysis, accession number in GenBank, and minimum inhibitory concentration (MIC) data of 75 UM isolates of dematiaceous fungi. (DOCX) [file pone.0104352.s001.docx]

**Table S1.** Isolate strain, clinical source, morphological identity, molecular identity based on phylogenetic analysis, accession number in GenBank and Minimum Inhibitory Concentration (MIC) data of 75 UM isolates of dematiaceous fungi.

| **No.** | **Isolate ID** | **Isolation source** | **Morphological Identity** | **Molecular Identity** | **Accession Number** | **MIC (µg/mL)** | | | | | | | |
| --- | --- | --- | --- | --- | --- | --- | --- | --- | --- | --- | --- | --- | --- |
|  |  |  |  |  |  | **AMB^b^** | **KTC^c^** | **FLC^d^** | **ITC^e^** | **VRC^f^** | **PSC^g^** | **ANID^h^** | **CAS^i^** |
| **1.** | **UM 75** | Nail | *Alternaria* sp. | *Alternaria arborescens* | JX966632 | 0.064 | 0.125 | >256 | 0.032 | 0.25 | 0.064 | 0.19 | 0.008 |
| **2.** | **UM 221** | Nasopharyngeal secretion | n.d^a^ | *Ascomycota* sp. | JX966643 | 0.75 | 1.5 | >256 | 8 | 0.023 | 0.5 | 0.5 | >32 |
| **3.** | **UM 235** | Nail | *Chaetomium* sp. | *Chaetomium brasiliense* | JX966545 | 12 | 0.38 | 4 | 0.5 | 0.012 | 0.75 | 0.19 | 0.25 |
| **4.** | **UM 265** | Nail | *Cladosporium* sp. | *Cladosporium cladosporioides* | JX966584 | 4 | 0.38 | >256 | 0.5 | 0.75 | 0.125 | 0.002 | >32 |
| **5.** | **UM 315** | Skin scraping | *Cladosporium* sp. | *Cladosporium cladosporioides* | JX966581 | 0.38 | 0.25 | >256 | 0.094 | 0.25 | 0.023 | 0.008 | >32 |
| **6.** | **UM 318** | Nail | *Cladosporium* sp. | *Cladosporium cladosporioides* | JX966582 | 0.38 | 0.125 | >256 | 0.064 | 0.125 | <0.002 | 0.023 | >32 |
| **7.** | **UM 77** | Blood | *Cladosporium* sp. | *Cladosporium cladosporioides* | JX966583 | 0.75 | 0.38 | <0.016 | 0.016 | 0.19 | 0.008 | 0.047 | >32 |
| **8.** | **UM 155** | Skin scraping | *Cladosporium* sp. | *Cladosporium dominicanum* | JX966585 | 12 | 0.38 | >256 | 2 | 1 | 0.25 | 0.003 | 0.125 |
| **9.** | **UM 165** | Nail | *Cladosporium* sp. | *Cladosporium sphaerospermum* | JX966566 | 3 | 0.005 | 32 | 0.047 | 0.016 | 0.008 | 0.004 | 0.016 |
| **10.** | **UM 187** | Skin lesion Swab | *Cladosporium* sp. | *Cladosporium sphaerospermum* | JX966577 | 1.5 | 0.023 | >256 | 0.016 | 0.064 | 0.012 | 0.032 | 0.016 |
| **11.** | **UM 225** | Skin scraping | *Cladosporium* sp. | *Cladosporium sphaerospermum* | JX966565 | 12 | 0.008 | 32 | 0.032 | 0.008 | 0.006 | 0.003 | 0.094 |
| **12.** | **UM 245** | Skin lesion Swab | *Cladosporium* sp. | *Cladosporium sphaerospermum* | JX966579 | 1.5 | 0.75 | >256 | 0.38 | 0.25 | 0.125 | <0.002 | 0.012 |
| **13.** | **UM 269** | Skin scraping | *Cladosporium* sp. | *Cladosporium sphaerospermum* | JX966564 | 2 | 0.047 | 16 | 0.032 | 0.094 | 0.023 | 0.008 | >32 |
| **14.** | **UM 333** | Skin scraping | *Cladosporium* sp. | *Cladosporium sphaerospermum* | JX966574 | 1 | 0.094 | 8 | 0.38 | 0.094 | 0.094 | 8 | 0.5 |
| **15.** | **UM 349** | Blood | *Cladosporium* sp. | *Cladosporium sphaerospermum* | JX966578 | 3 | 0.25 | >256 | 0.125 | 0.25 | 0.016 | 0.006 | <0.002 |
| **16.** | **UM 350** | Blood | *Cladosporium* sp. | *Cladosporium sphaerospermum* | JX966573 | 0.023 | 1 | 0.094 | 0.012 | 0.016 | 0.047 | 0.003 | <0.002 |

Table S1 cont.

| **No.** | **Isolate ID** | **Isolation source** | **Morphological Identity** | **Molecular Identity** | **Accession Number** | **MIC (µg/mL)** | | | | | | | |
| --- | --- | --- | --- | --- | --- | --- | --- | --- | --- | --- | --- | --- | --- |
|  |  |  |  |  |  | **AMB^b^** | **KTC^c^** | **FLC^d^** | **ITC^e^** | **VRC^f^** | **PSC^g^** | **ANID^h^** | **CAS^i^** |
| **17.** | **UM 351** | Blood | *Cladosporium* sp. | *Cladosporium sphaerospermum* | JX966575 | <0.002 | 0.008 | >256 | <0.002 | 0.5 | 0.006 | 0.012 | 0.003 |
| **18.** | **UM 352** | Skin scraping | *Cladosporium* sp. | *Cladosporium sphaerospermum* | JX966567 | 1 | 0.012 | 6 | 0.012 | 0.047 | 0.003 | 0.012 | <0.002 |
| **19.** | **UM 353** | Nail | *Cladosporium* sp. | *Cladosporium sphaerospermum* | JX966570 | 0.5 | 0.064 | 4 | 0.016 | 0.19 | 0.047 | 0.004 | 0.003 |
| **20.** | **UM 354** | Nail | *Cladosporium* sp. | *Cladosporium sphaerospermum* | JX966569 | >32 | 0.125 | >256 | 1 | 0.125 | 0.047 | <0.002 | 0.047 |
| **21.** | **UM 67** | Blood | *Cladosporium* sp. | *Cladosporium sphaerospermum* | JX966572 | 0.5 | 0.75 | >256 | 0.004 | 0.5 | 0.016 | 0.006 | 0.094 |
| **22.** | **UM 68** | Blood | *Cladosporium* sp. | *Cladosporium sphaerospermum* | JX966571 | 0.25 | 0.19 | >256 | 0.012 | 0.75 | 0.012 | 0.006 | 0.032 |
| **23.** | **UM 76** | Blood | *Cladosporium* sp. | *Cladosporium sphaerospermum* | JX966568 | 0.5 | 1.5 | >256 | 0.5 | 2 | 0.064 | 0.064 | >32 |
| **24.** | **UM 843** | Blood | *Cladosporium* sp. | *Cladosporium sphaerospermum* | JX966576 | 3 | 0.008 | 2 | 0.023 | 0.5 | 0.023 | 0.064 | 0.23 |
| **25.** | **UM 226** | Skin scraping | *Bipolaris* sp. | *Bipolaris papendorfii*/ *Cochliobolus geniculatus* | JX966599 | 0.012 | 0.032 | 1 | 0.012 | 0.023 | 0.006 | 0.004 | 0.094 |
| **26.** | **UM 183** | Skin lesion Swab | *Bipolaris* sp. | *Cochliobolus geniculatus* | JX966589 | <0.002 | 0.064 | 1.5 | 0.004 | 0.016 | 0.032 | 0.003 | <0.002 |
| **27.** | **UM 217** | Skin scraping | *Bipolaris* sp. | *Cochliobolus geniculatus* | JX966590 | 0.047 | 0.5 | 8 | 0.032 | 0.032 | 0.012 | 0.006 | 0.094 |
| **28.** | **UM 236** | Skin lesion Swab | *Bipolaris* sp. | *Cochliobolus geniculatus* | JX966588 | 0.094 | 0.25 | 8 | 0.125 | 0.064 | 0.023 | 0.016 | 0.75 |
| **29.** | **UM 191** | Skin lesion Swab | *Bipolaris* sp. | *Cochliobolus hawaiiensis* | JX966615 | 0.016 | 0.032 | 1.5 | 0.012 | 0.023 | 0.008 | 0.25 | 0.064 |
| **30.** | **UM 164** | Tissue biopsy | *Curvularia* sp. | *Cochliobolus lunatus* | JX966621 | 0.032 | 0.75 | 8 | 0.38 | 0.125 | 0.094 | 0.006 | 0.064 |
| **31.** | **UM 189** | Nail | *Curvularia* sp. | *Cochliobolus lunatus* | JX966607 | 0.064 | 0.19 | 6 | 0.023 | 0.094 | 0.016 | 0.064 | 0.25 |
| **32.** | **UM 201** | Blood | *Curvularia* sp. | *Cochliobolus lunatus* | JX966625 | 0.023 | 0.19 | 1 | 0.38 | 0.016 | 0.016 | 0.006 | 0.023 |

Table S1 cont.

| **No.** | **Isolate ID** | **Isolation source** | **Morphological Identity** | **Molecular Identity** | **Accession Number** | **MIC (µg/mL)** | | | | | | | |
| --- | --- | --- | --- | --- | --- | --- | --- | --- | --- | --- | --- | --- | --- |
|  |  |  |  |  |  | **AMB^b^** | **KTC^c^** | **FLC^d^** | **ITC^e^** | **VRC^f^** | **PSC^g^** | **ANID^h^** | **CAS^i^** |
| **33.** | **UM 239** | Skin scraping | *Curvularia* sp. | *Cochliobolus lunatus* | JX966622 | 0.032 | 0.75 | >256 | 0.064 | 0.5 | 0.19 | 0.004 | 0.047 |
| **34.** | **UM 272** | Skin scraping | *Curvularia* sp. | *Cochliobolus lunatus* | JX966623 | 0.032 | 0.19 | 12 | 0.5 | 1 | 0.064 | 0.125 | 0.5 |
| **35.** | **UM 296** | Nail | *Curvularia* sp. | *Cochliobolus lunatus* | JX966624 | 0.064 | 0.023 | 4 | 0.064 | 0.008 | 0.016 | 0.008 | 0.094 |
| **36.** | **UM 313** | Skin scraping | *Curvularia* sp. | *Cochliobolus lunatus* | JX966628 | 0.003 | 0.012 | 0.38 | <0.002 | 0.008 | 0.003 | <0.002 | 0.023 |
| **37.** | **UM 327** | Skin scraping | *Bipolaris* sp. | *Cochliobolus lunatus* | JX966626 | 0.032 | 0.032 | 0.75 | 0.016 | 0.032 | 0.016 | <0.002 | 0.047 |
| **38.** | **UM 248** | Nail | *Bipolaris* sp. | *Cochliobolus verruculosus* | JX966629 | 0.047 | 0.064 | 1.5 | 0.004 | 0.016 | 0.012 | 0.006 | 0.016 |
| **39.** | **UM 193** | Skin scraping | *Curvularia* sp. | *Curvularia affinis* | JX966612 | 0.032 | 0.016 | 4 | 0.004 | 0.032 | 0.032 | 0.004 | 0.125 |
| **40.** | **UM 262** | Skin scraping | *Curvularia* sp. | *Curvularia affinis* | JX966602 | 0.023 | 0.094 | 3 | 0.016 | 0.094 | 0.006 | 0.012 | 0.064 |
| **41.** | **UM 69** | Skin scraping | *Curvularia* sp. | *Curvularia affinis* | JX966610 | 0.047 | 0.38 | 3 | 0.047 | 0.047 | 0.016 | 0.006 | 0.19 |
| **42.** | **UM 297** | Nail | *Curvularia* sp. | *Curvularia eragrostidis* | JX966616 | 0.19 | 1 | >256 | 1 | 0.38 | 0.38 | 0.012 | 0.38 |
| **43.** | **UM 1020** | Blood | *Daldinia* sp. | *Daldinia eschscholzii* | JX966563 | 0.125 | 0.003 | <0.016 | <0.002 | <0.002 | 0.004 | <0.002 | 3 |
| **44.** | **UM 1400** | Skin scraping | *Daldinia* sp. | *Daldinia eschscholzii* | JX966561 | <0.002 | 0.012 | 1.5 | 0.064 | <0.002 | <0.002 | <0.002 | 0.008 |
| **45.** | **UM 230** | Nail | *Daldinia* sp. | *Daldinia eschscholzii* | JX966562 | 0.19 | 0.064 | 6 | 0.5 | 0.125 | 0.064 | 0.094 | 2 |
| **46.** | **UM 233** | Skin scraping | *Exophiala* sp. | *Exophiala dermatitidis* | JX966558 | 0.5 | 0.5 | 24 | 0.38 | 0.032 | 0.094 | >32 | >32 |
| **47.** | **UM 247** | Skin lesion Swab | *Exophiala* sp. | *Exophiala spinifera* | JX966556 | 0.047 | 0.047 | 2 | 0.064 | 0.032 | 0.018 | >32 | >32 |
| **48.** | **UM 162** | Nail | *Exophiala sp.* | *Exophiala xenobiotica* | JX966559 | 0.064 | 0.032 | 6 | <0.002 | 0.016 | 0.016 | 0.38 | >32 |
| **49.** | **UM 241** | Tissue biopsy | *Exserohilum sp.* | *Exserohilum rostratum* | JX966631 | 0.047 | 0.5 | 32 | 0.125 | 0.25 | 0.012 | 0.008 | 0.064 |
| **50.** | **UM 238** | Skin scraping | n.d^a^ | *Herpotrichiellaceae* sp. | JX966560 | 0.064 | <0.002 | 1.5 | <0.002 | <0.002 | <0.002 | <0.002 | <0.002 |

Table S1 cont.

| **No.** | **Isolate ID** | **Isolation source** | **Morphological Identity** | **Molecular Identity** | **Accession Number** | **MIC (µg/mL)** | | | | | | | |
| --- | --- | --- | --- | --- | --- | --- | --- | --- | --- | --- | --- | --- | --- |
|  |  |  |  |  |  | **AMB^b^** | **KTC^c^** | **FLC^d^** | **ITC^e^** | **VRC^f^** | **PSC^g^** | **ANID^h^** | **CAS^i^** |
| **51.** | **UM 202** | Nail | *Neoscytalidium* sp. | *Neoscytalidium dimidiatum* | JX966541 | 0.023 | 0.19 | 3 | 12 | 0.064 | 0.125 | 0.006 | 0.008 |
| **52.** | **UM 231** | Nail | *Neoscytalidium* sp. | *Neoscytalidium dimidiatum* | JX966544 | 0.023 | 0.008 | 0.125 | 0.003 | 0.012 | 0.003 | 0.02 | 0.125 |
| **53.** | **UM 249** | Nail | *Neoscytalidium* sp. | *Neoscytalidium dimidiatum* | JX966543 | 0.008 | 0.006 | 16 | 0.032 | 0.023 | 0.047 | 0.006 | 0.094 |
| **54.** | **UM 73** | Skin lesion Swab | *Neoscytalidium* sp. | *Neoscytalidium dimidiatum* | JX966539 | 0.064 | 0.19 | 1.5 | 0.004 | 0.016 | 0.008 | 0.012 | 0.125 |
| **55.** | **UM 153** | Nail | *Neoscytalidium* sp. | *Neoscytalidium dimidiatum*/ *Neoscytalidium novaehollandiae* | JX966542 | 0.032 | 0.064 | 3 | 0.032 | <0.002 | 0.047 | <0.002 | 0.094 |
| **56.** | **UM 224** | Nail | *Neoscytalidium* sp. | *Neoscytalidium dimidiatum*/ *Neoscytalidium novaehollandiae* | JX966540 | 0.032 | 0.75 | 0.19 | 1 | 0.003 | 0.008 | 0.064 | 0.047 |
| **57.** | **UM 157** | Skin scraping | *Nigrospora* sp. | *Nigrospora oryzae* | JX966548 | 0.064 | >32 | >256 | >32 | 12 | >32 | 24 | >32 |
| **58.** | **UM 160** | Skin scraping | *Nigrospora* sp. | *Nigrospora oryzae* | JX966549 | 0.012 | 4 | >256 | >32 | 4 | >32 | 16 | >32 |
| **59.** | **UM 244** | Skin scraping | *Nigrospora* sp. | *Nigrospora oryzae* | JX966547 | 0.006 | 0.047 | 6 | 4 | 8 | 0.094 | 1.5 | 2 |
| **60.** | **UM 299** | Nail | Mycelia sterilia | *Nigrospora oryzae* | JX966550 | 0.064 | 0.006 | 1.5 | 0.004 | 0.008 | 0.012 | >32 | >32 |
| **61.** | **UM 314** | Skin scraping | *Ochroconis* sp. | *Ochroconis constricta* | JX966647 | >32 | >32 | >256 | 0.125 | 0.75 | 0.25 | 0.008 | 0.125 |
| **62.** | **UM 324** | Skin scraping | *Ochroconis* sp. | *Ochroconis constricta* | JX966645 | >32 | 0.125 | >256 | 0.25 | 0.125 | 0.094 | 0.012 | 0.094 |
| **63.** | **UM 326** | Skin scraping | *Ochroconis sp.* | *Ochroconis constricta* | JX966646 | >32 | 0.38 | >256 | 0.047 | 0.125 | 0.064 | 0.008 | 0.125 |
| **64.** | **UM 329** | Skin scraping | *Ochroconis sp.* | *Ochroconis constricta* | JX966644 | >32 | 0.38 | >256 | 0.125 | 0.19 | 0.047 | 0.002 | 0.094 |
| **65.** | **UM 228** | Skin lesion Swab | *Phoma* sp. | *Phoma gardeniae* | JX966640 | 1.5 | 0.125 | 4 | 0.064 | 0.19 | 0.023 | <0.002 | 0.032 |

Table S1 cont.

| **No.** | **Isolate ID** | **Isolation source** | **Morphological Identity** | **Molecular Identity** | **Accession Number** | **MIC (µg/mL)** | | | | | | | |
| --- | --- | --- | --- | --- | --- | --- | --- | --- | --- | --- | --- | --- | --- |
|  |  |  |  |  |  | **AMB^b^** | **KTC^c^** | **FLC^d^** | **ITC^e^** | **VRC^f^** | **PSC^g^** | **ANID^h^** | **CAS^i^** |
| **66.** | **UM 298** | Nail | *Phoma* sp. | *Phoma gardeniae* | JX966638 | 1 | 0.094 | 24 | 0.25 | 0.094 | 0.094 | 0.008 | 0.094 |
| **67.** | **UM 223** | Nail | *Phoma* sp. | *Phoma multirostrata* | JX966636 | 0.75 | 0.064 | 8 | 0.094 | 0.064 | 0.19 | 0.006 | 0.006 |
| **68.** | **UM 186** | Nail | Mycelia sterilia | *Phoma multirostrata*/ *Epicoccum sorghi* | JX966634 | 0.023 | 0.25 | 0.19 | 0.19 | 0.125 | 0.125 | 0.008 | 0.016 |
| **69.** | **UM 255** | Nail | Mycelia sterilia | *Phoma multirostrata*/ *Epicoccum sorghi* | JX966635 | 0.016 | 0.19 | >256 | 0.25 | 0.064 | 0.19 | <0.002 | 0.064 |
| **70.** | **UM 254** | Nail | Mycelia sterilia | *Phomopsis* sp. /*Diaporthe arctii* | JX966551 | 0.023 | 0.008 | >256 | >32 | 0.19 | 0.004 | 0.047 | 0.094 |
| **71.** | **UM 256** | Skin scraping | *Phoma* sp. | *Pyrenochaeta unguis-hominis* | JX966641 | 1.5 | >32 | >256 | >32 | >32 | >32 | <0.002 | >32 |
| **72.** | **UM 212** | Tissue biopsy | *Exophiala* sp. | *Rhinocladiella atrovirens* | JX966554 | 8 | 1.5 | >256 | 0.38 | 0.75 | 0.047 | >32 | >32 |
| **73.** | **UM 234** | Skin scraping | *Exophiala* sp. | *Rhinocladiella atrovirens* | JX966555 | 8 | 1 | >256 | 0.19 | 0.5 | 0.008 | 0.75 | 0.25 |
| **74.** | **UM 1110** | Nasopharyngeal secretion | n.d^a^ | *Stagonospora* sp./ *Septoria arundinacea* | JX966633 | 0.19 | 0.125 | 3 | 0.25 | 0.19 | 0.023 | 0.75 | <0.002 |
| **75.** | **UM 259** | Skin scraping | Mycelia sterilia | *Stagonospora* sp./ *Septoria arundinacea* | JX966642 | 0.19 | 0.016 | 1 | 0.016 | 0.012 | 0.003 | <0.002 | >32 |

^a^No identifiable morphological features

^b^Amphotericin B

^c^Ketoconazole

^d^Fluconazole

^e^Itraconazole

^f^Voriconazole

^g^Posaconazole

^h^Anidulafungin

^i^Caspofungin
